# Analysis of Gender Differences in the Impact of Taxation and Taxation Structure on Cigarette Consumption in 17 ITC Countries

**DOI:** 10.3390/ijerph16071275

**Published:** 2019-04-10

**Authors:** Anh Ngo, Geoffrey T. Fong, Lorraine V. Craig, Ce Shang

**Affiliations:** 1 Oklahoma Tobacco Research Center, Stephenson Cancer Center, the University of Oklahoma Health Sciences Center; Oklahoma, OK 73104, USA; Anh-Ngo@ouhsc.edu; 2 Department of Psychology, University of Waterloo, Waterloo, ON N2L 3G1, Canada geoffrey.fong@uwaterloo.ca (G.T.F.); lvcraig@uwaterloo.ca (L.V.C.); 3 School of Public Health and Health Systems, University of Waterloo, Waterloo, ON N2L 3G1, Canada; 4 Ontario Institute for Cancer Research, Toronto, ON M5G 0A3, Canada

**Keywords:** taxation, taxation structure, gender differences, cigarette consumption

## Abstract

Although increasing taxes has been established as the most effective tobacco control policy, it is not clear whether these policies reduce cigarette consumption equally among women and men. In this study, we examine whether the association between taxation/taxation structure and cigarette consumption differs by gender. The data is from the International Tobacco Control Policy Evaluation (ITC) Projects in 17 countries. Cigarette consumption was measured by gender for each ITC country. Generalized estimating equations (GEE) were employed to investigate gender differences in the association between cigarette consumption and tax structures, while controlling for time-variant demographic characteristics such as unemployment rates, proportions of adults, and percent of female population. Tiered tax structures are associated with higher cigarette consumption among both males and females. Female smokers are more responsive to an average tax increase than male smokers. Among males, higher ad valorem share in excise taxes is associated with lower cigarette consumption, but it is not the case for females. Females may not be as responsive to the prices raised by ad valorem taxes, despite being responsive to average taxes, suggesting that smokers by gender may face different prices.

## 1. Introduction

Tobacco use is one of the leading causes of non-communicable diseases worldwide [1]. According to the World Health Organization, there are 1.1. billion smokers worldwide [2]. Globally, over 40% of men are estimated to smoke while approximately 10% of women do [3,4]. While in high-income countries, women smoke at almost the same rate as men, in low and middle income countries (LMICs), they smoke at a much lower rate [4]. In 2016, 17.5% of US adult males and 13.5% of US adult females smoked cigarettes [5]. On the other hand, in China (2015) and Thailand (2016), while approximately 48% and 39% of men smoked respectively, only 1.9% of women smoked (Table 1). The World Health Organization (WHO) Framework Convention on Tobacco Control (FCTC) obligates 181 countries to implement a comprehensive set of policy measures, such as excise taxes, warning labels, and smoke-free policies, to curb tobacco use. As a result, tobacco control policies in many countries have been strengthened and there is growing evidence that these policies are effective in reducing tobacco use [6,7,8].

Increasing taxes and prices is the most effective tobacco control measure [9]. It is estimated that a 10% increase in the real price of cigarettes would decrease smoking by 4% in high income countries and by 8% in LMICs [10]. While a set of ‘non-price’ measures would decrease the number of smokers in 1995 by 23 million and prevent 5 million premature tobacco related deaths, raising cigarette prices by 10% would potentially lead to 42 million smokers quitting and avert 10 million deaths [10]. In particular, recent studies have suggested that, beyond increasing average taxes, tax structures play a role in price variability and thus tax avoidance opportunities [11,12,13]. To be specific, tax structure is defined in the frame that raising taxes depends not only on what basis taxes are levied but also on how the tax rates are determined [11,12,13,14]. Currently, there are six different types of tax structures: (1) specific uniform (quantity based, a single rate), (2) specific tiered (quantity based, multiple rates), (3) ad valorem uniform (value based, a single rate), (4) ad valorem tiered (value based, multiple rates), (5) mixed uniform (both quantity based and value based, single rates), and (6) mixed tiered (both quantity based and value based, multiple rates) [13]. While a specific uniform tax structure is the simplest form with one single tax rate based on quantities, the others are more complicated with different tax rates for different products. In some countries, since tax rates may vary by different product characteristics such as stick length and price tiers, tobacco manufacturers could strategically bypass taxes, set prices, or design products to take advantage of complicated tax structures [15].

Despite progress, it is uncertain whether tobacco control policies are equally effective among populations with various sociodemographic characteristics, such as gender [16]. For example, although increasing taxes has been established as the most effective tobacco control policy [9], it is not clear whether these policies reduce cigarette consumption equally among women and men. In addition, gender differences in exploring tax avoidance opportunities may further influence the effectiveness of taxation policies. The limited evidence from high-come countries (HICs) is mixed regarding whether female smokers are more or less responsive to taxes and prices compared to their male counterparts, while being more likely to engage in tax avoidance behaviors [17,18]. Farrelly et al. (2001) [19] investigated the impact of cigarette price increases by gender, income, race, and ethnicities in the US, and documented that women were more responsive to increased prices than men, with a price elasticity of −0.32 for women and −0.18 for men. Likewise, Stehr (2007) [17] found that US women were two times more responsive to cigarette tax changes than men. On the other hand, Hersch (2000) [20], Chaloupka (1990) [21], and Lewit and Coate (1981) [22] documented the opposite results. Cornelius et al (2015) [18] investigated trends in cigarette prices and purchasing patterns among a group of US adult smokers from 2002 to 2012 and further found that compared to males, females were more likely to engage in tax avoidance behaviors by buying cigarettes by carton or in multipacks and in locations associated with tax avoidance such as duty free shops and Indian reservations [18].

Previous studies have shown that weight-related concerns such as gaining weight after quitting discourage smokers to quit and make quit attempts [23,24,25,26]. Compared to males, females are more likely to express weight concerns and less likely to make a quit attempt in response to policies if they believe smoking can help regulate weight [27]. These findings suggest that women may respond to prices differently from men. Since women believe that smoking can help them to control weight, they may be unresponsive to increases in cigarette prices and are more likely to initiate smoking or continue to smoke than men when they experience a weight gain [28]. Women who hold such a belief have also been found less responsive to increasing prices than those who do not [29]. Specifically, in LMICs, tobacco use prevalence can differ significantly by gender and female users may prefer products other than cigarettes (e.g., smokeless tobacco in India), which are understudied [1]. With smoking-related burden disproportionately shifted to LMICs and lower socioeconomic (SES) populations in HICs, understanding how tobacco control policies such as tax policies perform in reducing tobacco use among vulnerable populations will provide important insights for improving the effectiveness of policies [1].

A growing number of studies have documented an association between complicated tax structures and a greater price gap between higher- and lower- priced products, and thus more opportunities for smokers to switch to cheaper products to avoid taxes in response to a tax increase [13,14]. Recent evidence from the International Tobacco Control (ITC) Policy Evaluation Project further links complicated tax structures with higher cigarette consumption data from 17 ITC countries and found that changing from a specific to an ad valorem structure was associated with higher cigarette consumption in HICs, whereas a change from a uniform to tiered structure is associated with higher cigarette consumption in LMICs [30].

Although previous studies have examined gender differences in the effect of cigarette taxes on smoking, the evidence is mixed and mainly from high-income countries. In addition, to the best of our knowledge, no studies have investigated gender differences in the impact of taxation and taxation structure on cigarette consumption. Thus, more research is needed to better understand gender differences in tobacco control policy effectiveness from a broader context, focusing on taxation. Stronger evidence of taxation policy effectiveness among females, in comparison to males, will shed light on how to enhance the overall impact of taxation policies. In this study, we expanded the existing ITC analyses on taxation and taxation structure to examine whether their associations with cigarette consumption in daily smokers differ by gender, and provided evidence of a broader scope with datasets from multiple countries.

## 2. Materials and Methods

### 2.1. Cigarette Consumption

Cigarette consumption was measured by gender for each ITC country, using the logarithm of the number of cigarettes that an average female (or male) smoker smoked per day. These data were derived from self-reported consumption questions in the ITC longitudinal surveys from 17 countries. The surveys were designed to systematically evaluate the psychosocial and behavioral impact of tobacco control policies under the WHO FCTC [11,31]. The surveys were conducted through telephone, face-to-face, and online interviews, and provided the information on self-reported cigarette consumption of the population over time. All waves of the surveys from 2002 to 2013 in 17 countries were used to examine gender differences in the association between tax structure and cigarette consumption. The following countries were included in the study: US, UK, Australia, Canada, the Netherlands, Germany, France, Republic of Korea, Mexico, Brazil, Uruguay, Mauritius, India, Bangladesh, China, Thailand, and Malaysia.

### 2.2. Cigarette Excise Tax Structures

The data on cigarette excise tax structures were gathered for each country over time from various sources. Table 9.1.0 of the 2013 WHO Report on the Global Tobacco Epidemic is the primary source. The table summarizes the prices (per 20-cigarette pack) of the most popular brands and the shares of ad valorem and/or specific tax in those prices [31,32]. Excise Duty Tables by the European Commission, the WHO country reports, the Technical Manual on Tobacco Tax Administration, Global data’s country reports, and Euromonitor International’s country reports were also used as sources of the tax data. The tax information from these data sources was described in details in a previous study [13].

Tax structures were measured using a dummy variable for any tiered rates and a continuous variable ranging from 0 to 100 to indicate the components or shares of ad valorem bases [13]. While 0 represented the specific structure, 100 represented the ad valorem structure, and any number in-between represented a mixed structure. A dummy variable indicating whether a country had excise taxes imposed at the local level was constructed for India, the US, and Canada. To further control for the constraint that the European Union (EU) imposed on member countries to implement a mixed tax structure, a dummy variable is constructed.

### 2.3. Cigarette Excise Taxes

We gathered the data on annual excise taxes in 2010 dollars using similar sources. Table 9.1.0 of the 2013 WHO Report on the Global Tobacco Epidemic provided the tax rate information from 2008 to 2012 for most countries except for the US, Canada, Australia, and EU countries [31]. Thus, the excise tax information of EU countries from 2002 onwards was from the Excise Duty Tables by the European Commission, and the information of Australia was from the Australian Taxation Office. On the other hand, the information on federal and average state excise taxes in the US came from Tax Burden on Tobacco by Orzechowski and Walker. Finance and Treasury Board of Canada published Other Comparative Tax Rates, which was a population-weighted average of the federal and provincial taxes. The tax rate data for the rest of countries came from the Euromonitor reports and WHO periodic reports on global tobacco epidemic.

### 2.4. Demographic Characteristics

The information on country-year level demographic characteristics was from the World Bank’s World Development Indicators Database [33]. The information gathered was gross domestic product (GDP) per capita in international dollars, unemployment rates, the percentage of population aged 15 and over, and the percent of female population. A dummy variable indicating a high-income country was also constructed using the World Bank’s income group classification when surveys were conducted. No countries changed their income group classification during the study period.

All data sources were linked together using country and year identifiers to compile the final analytical sample. Since some of the ITC survey waves were conducted across two calendar years [13], the calendar year was randomly assigned to the wave in these cases. Cigarette consumption, excise taxes, and GDP per capita were in log form to derive the tax and income elasticities.

### 2.5. Methodology

Following Shang et al. (2015) [13], generalized estimating equations (GEE) were employed to investigate the association between cigarette consumption and tax structures, using an identity link, inversed Guassian family and exchangeable correlation setting. By utilizing GEE, we accounted for any intertemporal correlations. Previous studies have shown that tax structures are associated with lower average prices and greater price variability [13,14]. Thus, we employed two different specifications to better capture the association between tax structures and cigarette consumption. In the first specification, we regressed cigarette consumption on tax structure measures and countries’ sociodemographic characteristics without controlling for average taxes. Alternatively, we controlled for the same covariates and average taxes in the second specification. The equation employed in the second specification—a more flexible approach is as follows.

Consumption_ijt_ = a0 + a1 tax_jt_ + a2 tax_jt_ × Male_i_ + a4 ad valorem_jt_ + a5 ad valorem_jt_ × Male_i_ + a6 tiered_jt_+ a7 tiered_jt_ × Male_i_ + a8 Male_i_+ a9 income_jt_ + other control_jt_ + year_t_ (1)

Consumption_ijt_ denotes the average number of cigarettes smoked per day by males and females (i) in a country j at time t. The male dummy variable equals 1 if the average consumption was aggregated based on males’ reports and 0 if based on females’ reports. To directly obtain the tax and income elasticities, cigarette consumption, excise taxes, and income (GDP per capita) were in log form. Other covariates included in the regression are time-variant demographic characteristics such as unemployment rates, proportions of adults, and percent of female population. A dummy variable indicating excise taxes imposed at the local level in India, the US, and Canada and a dummy variable indicating EU member countries were also included in the regression. Year indicators were included to account for time-invariant factors that may affect cigarette use. 

The coefficients of interest are on the interaction terms. These interaction coefficients capture the gender difference in individuals’ responses to taxes. For instance, in the context that taxes and consumption are negatively associated (a1 < 0), a positive significant sign of a2 indicates that females are more responsive to taxes than males. On the other hand, in the context when a higher share of ad valorem taxes and tiered rates are associated with more consumption (a4 and a6 > 0), a negative significant sign of the coefficient on tax structure variables (a5 and a7) suggests that females are more likely to take advantage of tax avoidance opportunities and thus less likely to reduce their cigarette consumption. All analyses were conducted using the command XTGEE in Stata SE V.14.1 (StataCorp, College Station, TX, USA).

## 3. Results

Table 1 presents the information on tax structure, share of ad valorem tax among total excise taxes, average cigarette consumption per day, and smoking prevalence in 17 countries in the analytical sample. As Table 1 indicates, six countries (35%) had specific uniform tax structures. Five countries (29%) had mixed uniform tax structures. The rest (36%) had specific tiered tax structures, mixed tiered tax structures, ad valorem, uniform, and ad valorem tiered tax structures. Two countries (Mexico and Brazil) changed their tax structures during the study period. While Mexico changed from an ad valorem uniform to a mixed uniform structure in 2009, Brazil switched to a mixed tiered system from a specific tiered in 2012 [13].

In terms of cigarette consumption, the average number of cigarettes smoked per day in HICs is quite high, with almost 17 cigarettes per day or even higher (more than four-fifths of a 20-stick pack) for the US, Canada, UK, Republic of Korea, and Australia. While smokers in China, on average, smoked around 17 sticks of cigarettes per day, smokers in India smoked around 6 sticks per day. Noticeably, Mexico experienced a decrease of 1.2 sticks (~17%) per day after changing to a mixed uniform structure. On the other hand, Brazil experienced a small increase of 0.31 sticks (~2%) per day after its tax structure changed to a mixed tiered system in 2012. 

Regarding smoking prevalence, in general, less than 20% of the population in HICs smoked, except for France (36%) and Germany (25%). For instance, smoking prevalence is 14% in the US in 2017, 13% in Canada in 2015, 17% and 19% in UK and Netherlands in 2017 respectively. As Table 1 further indicates, there is a large difference in smoking prevalence between males and females in LMICs. In 2015, while 47.6% of males in China smoked, smoking prevalence among women was only 1.8%. Likewise, in 2015, 43% of males in Malaysia smoked while smoking prevalence among females was only 1.4%. Similar gender differences occur in Thailand, Mexico, and Bangladesh.

Table 2 presents the information on the log cigarette consumption by gender and income groups. Overall, the log cigarette consumption of males and females in the whole sample are 2.603 and 2.547 respectively. There is no significant difference in the log consumption across gender (t = 1.01). In the HICs sample, females experienced a slightly higher log cigarette consumption of 2.815 than males with 2.715. On the other hand, the log cigarette consumption for males and females in the LMICs sample are 2.433 and 2.141 respectively.

Table 3 presents the gender difference in the association between tax structure and cigarette consumption in daily smokers across specifications. Model 1 included no controls for average taxes and HICs dummy. Model 2 further controlled for HICs dummy. Model 3 controlled for average taxes but not HICs dummy, and model 4 controlled for both. The results suggest that cigarette consumption elasticity was −0.2 to −0.3 (*p* < 0.05) for female smokers in the ITC countries, whereas male smokers are less responsive to taxes. As the coefficients on the tax structure variables (ad valorem and tiered) suggest, there is no significant association between female cigarette consumption and ad valorem tax structure. However, for males, there may be a negative association, given that the interaction term between males and ad valorem taxes remain negative across specifications. In addition, a tiered structure was associated with a 90–99% (*p* < 0.01) higher cigarette consumption among both genders, and there is no significant difference by gender. The income elasticities are from 0.4 to 0.6 across models, indicating that a 10% increase in GDP per capita was associated with a 4–6% increase (*p* < 0.01) in cigarette consumption. We also conducted stratified analyses by LMICs vs. HICs, but most coefficients become non-significant, which is likely due to the limited within country variation in a flexible model with many interactions.

## 4. Discussion

This study examined the gender difference in the association between taxation/taxation structures and cigarette consumption. We found that the tax elasticity of cigarette consumption is on average −0.3 among female smokers, whereas male smokers are less likely to respond to increasing taxes. This result is consistent with some of the existing studies, which find females to be more responsive to increasing prices than males [17,19].

Tax structure results indicate that tiered structures are associated with higher cigarette consumption with no significant gender difference. That is, both female and male smokers are equally likely to smoke more when tax structures are tiered. Moreover, although ad valorem tax shares are not significantly associated with female cigarette consumption, male consumption may be negatively associated with a higher ad valorem share. The possible explanation is that ad valorem taxes have the advantage over specific taxes in keeping up with inflation, particularly in areas with significant income growth. Therefore, depending on the study sample, ad valorem taxes may be negatively associated with cigarette consumption.

The combined evidence suggests that gender differences in tax effectiveness are complex, and likely dependent on which prices in the price distribution that different genders are facing. While female smokers are on average more responsive to taxes than males, they are not as responsive to ad valorem taxes as male smokers. The interpretation is subject to how we view and interpret ad valorem taxes. If ad valorem compared to specific taxes are more complicated and thus related to more consumption, this may suggest that there are more opportunities for females to engage in tax avoidance by switching to lower-priced products. However, given the negative association between ad valorem tax shares and male cigarette consumption, this is not likely the case. The more plausible interpretation is that ad valorem taxes can keep pace with income growth and inflation, and thus are linked with lower cigarette consumption among males. In this scenario, female smokers are not as likely as male smokers to be associated with lower cigarette consumption in response to ad valorem taxes. Lastly, a tiered structure has been identified as the least desirable structure since it cannot raise prices to keep pace with inflation like ad valorem taxes do, while providing room for tax avoidance. Female and male cigarette consumption are both higher under a tiered tax structure.

This study has some limitations. First, the data used in this study come from 17 countries and thus the results are not representative of all countries. In addition, the first wave of the surveys used in this study was collected in 2002, which is 17 years ago. This indicates the age of the data, which may affect generalizability of our results. In other words, our results only reflect the association between taxation/taxation structures and cigarette consumption during the study period 2002–2013. Second, due to the lack of data, we cannot control for the time-varying tobacco control environment in the models. In addition, other factors that can affect cigarette consumption may not be included in the models. Third, the information on cigarette consumption was gathered from aggregated self-reports and thus may contain self-report errors. Moreover, we measured cigarette consumption as the number of cigarette smoked per day. Thus, non-daily smokers were not included in this analysis. Lastly, in the regressions, we restricted the associations between GDP or unemployment rates and cigarette consumption to be the same by gender. It is certainly possible that the associations of other covariates with consumption may differ by gender. In the cases for income and unemployment rate, they also are highly correlated with tax structures and rates, which may pick up the gender differences in the associations between taxes and consumption. Therefore, we opted for a simple restricted model that allows us to more straightforwardly test and interpret the gender difference in the associations between tax and consumption.

Despite these limitations, our study contributes to the literature by adding much-needed evidence on the gender difference in tobacco control policy effectiveness, focusing on taxation. Our results indicate that tiered tax structures are associated with higher cigarette consumption among both males and females. Female smokers are more responsive to an average tax increase than male smokers. This indicates that raising cigarette taxes may reduce cigarette consumption among females. With the health risks related to smoking during pregnancy as well as its negative effects on infant health [37,38,39], policymakers may want to reduce smoking among females [19]. Among males, higher ad valorem share in excise taxes is associated with lower cigarette consumption. However, it is not the case for females. Females may not be as responsive to the prices raised by ad valorem taxes, despite being responsive to average taxes, suggesting that smokers by gender may face different prices. Thus, it would be better to maintain a simple tax structure instead of a complicated one.

Our study further offers the foundation for future large-scale projects that comprehensively study the effectiveness of tobacco control policies across different groups. Future research may link multiple datasets (ITC, GATS, GYTS, etc.) to examine policy effectiveness among other vulnerable populations, including females, youth, young adults, and low SES (LMIC) populations. In addition, future research may benefit from expanding our policy impact outcomes to examine smokeless tobacco use and dissect cigarettes into roll-your-own and manufactured products to test gender differences in tobacco use and policy effectiveness. As the literature suggests, females, youth, and low SES populations may be more likely to avoid taxes, switch products, or have barriers to behavioral changes (e.g., weight concerns), even if policies are implemented. Thus, more research is needed to examine policy effectiveness by accounting for these population differences in responses to policies.

## 5. Conclusions

To the best of our knowledge, our study is the first one to examine gender differences in the impact of taxation and taxation structure on cigarette consumption. We find that tiered tax structures are associated with higher cigarette consumption among both males and females. Female smokers are more responsive to an average tax increase than male smokers, indicating higher cigarette taxes as an important tool for decreasing cigarette consumption among females. Further, it may be better to maintain a simple tax structure instead of a complicated one. Future research may link multiple datasets to examine policy effectiveness among other vulnerable populations, including females, youth, young adults, and low SES (LMIC) populations. In addition, future research may benefit from expanding our policy impact outcomes to examine smokeless tobacco use and dissect cigarettes into roll-your-own and manufactured products to test gender differences in tobacco use and policy effectiveness.

## Figures and Tables

**Table 1:** Tax Structure and Average Daily Cigarette Consumption and Smoking Prevalence in 17 International Tobacco Control (ITC) countries.

Country	Tax Structure	Share of ad valorem tax	Average Consumption Per Day	Smoking Prevalence
Australia	Specific Uniform	0	17.32	14% (2016)
Canada		0	16.45	13% (2015)
Mauritius		0	9.25	40.1% for male, 3.3% for female (2015)
Republic of Korea		0	17.41	49.8% for male, 4.2% for female (2015)
USA		0	17.43	14% (2017)
Uruguay		0	15.53	26.7% for male, 19.4% for female (2015)
India	Specific tiered	0	5.68	20.4% for male, 1.9% for female (2015)
Brazil 2009		0	15.4	10.1% (2017)
France	Mixed uniform	89%	12.38	36% (2017)
Germany		41.60%	14.67	25% (2017)
Malaysia		25.10%	12.82	43% for male, 1.4% for female (2015)
Mexico 2010–2012		76%	5.97	20.8% for male, 6.6% for female (2015)
Netherlands		26.30%	14.69	19% (2017)
UK		43.20%	16.2	17% (2017)
Brazil 2012	Mixed tiered	22.10%	15.71	10.1% (2017)
China		94.10%	17.11	47.6% for male, 1.8% for female (2015)
Thailand	Ad valorem	100%	11.16	38.8% for male, 1.9% for female (2016)
Mexico 2006-2008	Uniform	100%	7.17	20.8% for male, 6.6% for female (2015)
Bangladesh	Ad valorem tiered	100%	10.11	39.8% for male, 0.7% for female (2015)

Note: Countries in the shadow have specific uniform and mixed uniform tax structures—the two most common tax structures during the study period. Other countries have either specific tiered, mixed tiered, ad valorem, uniform, or ad valorem tiered tax structure. Year that the data gathered in parentheses. The data on smoking prevalence were gathered from various online sources such as Tobacco Atlas [1], CBS News (US) [34], the 2016 National Drug Strategy Household Survey published by the Australian Institute of Health and Welfare [35], and Statista website for EU countries [36]. The information on countries’ tax structure and average daily consumption was gathered from Shang C, et al. (2018) [30].

**Table 2:** Cigarette consumption in daily smokers by gender and country income.

Samples	Whole Sample (*N* = 156)		HICs (*N* = 47)		LMICs (*N* = 31)	
Gender	Male	Female	Male	Female	Male	Female
Log (cigarette consumption)	2.603	2.547 ^a^	2.715	2.815	2.433	2.141
	(0.258)	(0.421)	(0.11)	(0.111)	(0.421)	(0.392)
Number of observations	78	78	47	47	31	31

Note: ^a^ The *t*-test results indicate no significant difference in the mean of log cigarette consumptions across gender (*t* = 1.010). Standard deviations in parentheses. HICs: high-income countries; LMICs: low and middle-income countries.

**Table 3:** Gender Differences in the Association between Tax Structure and Cigarette Consumption in Daily Smokers (*N* = 156).

Models	Model 1	Model 2	Model 3	Model 4
	(*N* = 156)	(*N* = 156)	(*N* = 156)	(*N* = 156)
Tax	-	-	−0.221 ***	−0.281 ***
			(0.059)	(0.061)
Tax × Male	-	-	0.167 *	0.172 *
			(0.085)	(0.083)
Ad valorem	0.056	0.055	0.039	0.01
	(0.132)	(0.141)	(0.118)	(0.129)
Ad valorem × Male	−0.326 *	−0.319 *	0.281 *	−0.263 ^+^
	(0.146)	(0.147)	(0.137)	(0.135)
Tiered	0.985 **	0.901 ***	0.737 *	0.504 ^+^
	(0.304)	(0.258)	(0.348)	(0.259)
Tired × Male	−0.047	−0.055	0.192	0.19
	(0.138)	(0.139)	(0.217)	(0.223)
Income	0.515 ***	0.413 **	0.564 ***	0.404 ***
	(0.134)	(0.119)	(0.138)	(0.107)
HICs dummy	No	Yes	No	Yes
Percent change in cigarette consumption in daily smokers in response to tax structure changes				
Changes to an ad valorem			0.016	
Tax structure			(0.047)	
Changes to a tiered one			0.293 *	
			(0.139)	

Note: HICs: High-income countries. Model 1 did not control for average taxes and HICs dummy. Model 2 controlled for HICs dummy. Model 3 controlled for average taxes, and model 4 controlled for both. All regressions controlled for dummies for European Union (EU), dummies for having local taxes, a gender dummy, and proportion of adults, percent of female population, year effects, and unemployment rates. The cigarette consumption, income, and tax measures are in log forms. Standard errors adjusted for inter-temporal correlations in parentheses. ^+^ *p* < 0.1, * *p* < 0.05, ** *p* < 0.01, *** *p* < 0.001. N: number of observations
